# Cellular Immunity Against BK Polyomavirus in Kidney Transplant Recipients: A Comprehensive Review

**DOI:** 10.1111/tid.14401

**Published:** 2024-11-05

**Authors:** Mohammed Al‐Talib, Anna Skaria, Siân Griffin

**Affiliations:** ^1^ Systems Immunity Research Institute Division of Infection and Immunity School of Medicine Cardiff University Cardiff UK; ^2^ Bristol Medical School University of Bristol Bristol UK; ^3^ Southmead Hospital North Bristol NHS Trust Bristol UK; ^4^ Department of Nephrology and Transplantation Cardiff and Vale University Health Board Cardiff UK

**Keywords:** BK Polyomavirus | BKPyVAN | cellular immunity | NK cells | T cells

## Abstract

BK polyomavirus (BKPyV) is an important opportunistic viral infection that complicates kidney transplantation. Uncontrolled viral replication may result in BKPyV‐associated nephropathy (BKPyVAN), a major cause of premature allograft damage and failure. In the continued absence of proven treatments, management relies on the empirical reduction of immunosuppression to facilitate an effective host immune response to clear the virus. This may be complicated by the risk of allograft rejection. There is compelling evidence that cellular immune responses are key to establishing control after viral reactivation. Measurable peripheral BKPyV‐specific T cell responses temporally correlate with declining viral loads and subsequent clearance. Conversely, these responses are delayed or absent in BKPyVAN. How these peripheral findings correspond to the intragraft response, and whether BKPyV‐specific T cells contribute to the immunopathology of BKPyVAN, remains poorly understood. Molecular techniques have provided some insights; however, these have been unable to fully discriminate BKPyVAN from cellular rejection to date. Furthermore, the contributions of components of innate cellular immunity, such as natural killer cells, are not known.

Herein, we review the role of cellular immunity in BKPyV infection in kidney transplant recipients. We discuss advances in the understanding of how the development, phenotype, and functionality of these responses may determine the balance between viral control and immunopathology, and how this knowledge is being translated into tools to prognosticate and guide individualized immunosuppression reduction. Lastly, we consider how further elucidation of these responses may inform the design of therapies that would revolutionize how BKPyV is managed after transplantation.

**Abbreviations**
BKPyV: BK polyomavirus
BKPyVAN: BK polyomavirus‐associated nephropathy
ELISPOT: enzyme‐linked immunospot
HLA: human leukocyte antigen
IFN−γ: interferon−γ
IL: interleukin
JCPyV: JC polyomavirus
KIR: killer‐cell immunoglobulin‐like receptor
KTR: kidney transplant recipient
MHC: major histocompatibility complex
NKG2D: Natural Killer Group 2D
PBMC: peripheral blood mononuclear cell
TCA: T‐cell assay
TCMR: T cell‐mediated rejection
TCR: T‐cell receptor
TNF‐α: tumor necrosis factor‐alpha
ULBP: UL16‐binding protein
VST: virus‐specific T cell

## 1 Introduction

The outstanding progress in solid organ transplantation since the advent of immunosuppressive therapies has come at the cost of placing the organ recipient at greater risk of malignancy and opportunistic infection. Among these complications, BK polyomavirus (BKPyV) represents a particularly challenging clinical diagnosis for kidney transplant recipients (KTRs) and clinicians. Up to 30% of KTRs will develop BKPyV DNAemia [1, 2, 3] and, of these, a proportion will progress to develop BKPyV‐associated nephropathy (BKPyVAN), with subsequent graft damage or failure. There are currently no specific therapies proven to treat BKPyV, and high‐quality evidence regarding how best to manage patients who develop BKPyV DNAemia and BKPyVAN is lacking. Although optimal strategies have not been defined, immunosuppression reduction results in eventual viral clearance in >90% of cases [4]. However, this is coupled with the risk of precipitating rejection of the transplant and graft damage or loss. The need for immunosuppression modification following BKPyV infection may further compromise long‐term graft outcomes in these patients [5].

**FIGURE 1:**
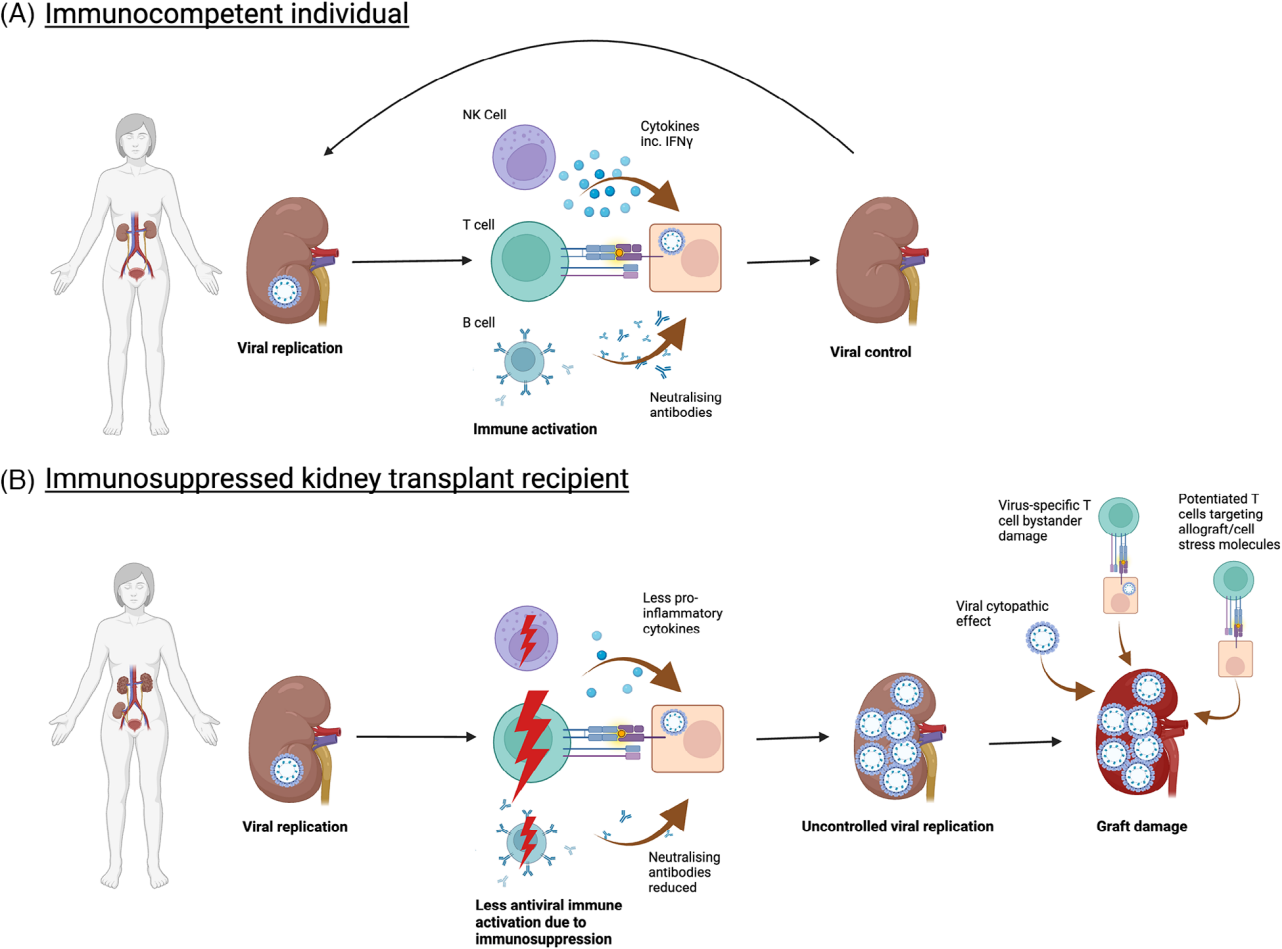
Mechanisms through which kidney transplant recipients are at risk of uncontrolled BK polyomavirus (BKPyV) replication and subsequent graft damage. (A) Intermittent episodes of viral replication trigger innate, humoral, and cellular immune responses that establish viral control. (B) Viral replication in the immunosuppressed kidney transplant recipient abrogates immune responses, resulting in uncontrolled viral replication and graft damage (BK polyomavirus‐associated nephropathy) through multiple mechanisms.

Primary infection in immunocompetent individuals is of no known clinical significance and appears to be ubiquitous, with epidemiological studies indicating seroprevalence rates exceeding 90% in children and adults [6, 7]. However, kidney transplantation routinely involves the administration of immunosuppressive therapies to abrogate T‐cell responses against the allograft and prevent rejection. Induction agents administered at the time of surgery include anti‐thymocyte globulin and alemtuzumab (anti‐CD52), which deplete T cells, or basiliximab (anti‐CD25) which inhibits T cell activation by preventing interleukin‐2 (IL‐2) signaling. Thereafter, maintenance immunosuppression typically involves a combination of a calcineurin inhibitor, antimetabolite, and/or steroid to prevent subsequent sensitization against graft alloantigens. Regardless of the agents used, the resultant profound T cell inhibition places KTRs at risk of developing BKPyV DNAemia and subsequently BKPyVAN. This has been associated with a higher “net state” of immunosuppression [8], with clinical resolution occurring in the majority following immunosuppression reduction, albeit coupled with the risk of rejection. Indeed, the incidence of BKPyV DNAemia is recognized to be greatest within the first few months after transplantation, when the immunosuppressive burden is at its highest [9, 10]. Furthermore, results from studies using functional T‐cell assays (TCAs) to quantify general cellular immune responsiveness suggest low TCA values may be associated with BKPyV infection [11, 12, 13, 14]. A myriad of host, donor, viral, and environmental factors likely predispose an individual to a greater risk of BKPyV DNAemia and/or BKPyVAN. Numerous risk factors have been variably identified and summarized in an excellent recent clinical review [15]. Importantly, many of these risk factors influence or condition the anti‐BKPyV cellular immune response (Figure 1).

So why is understanding the nature of the immune response elicited during BKPyV infection of KTRs important? The answer to this question arises from the challenges faced by clinicians managing BKPyV in KTRs. We currently cannot predict who is going to develop BKPyV DNAemia and, of those who do, we do not know who will spontaneously clear the virus [16, 17, 18] versus progress to BKPyVAN or require immunosuppression reduction. In those for whom a decision is made to reduce the immunosuppressive burden, how much is enough to control the infection while minimizing the risk of rejection? Where graft function is impaired, can we distinguish antiviral immune responses (where immunosuppression reduction facilitates viral control) from responses targeting graft alloantigens (requiring enhanced immunosuppression)? T cell‐mediated rejection (TCMR) shares many histological, and molecular [19], features with BKPyVAN, and the two may exist concomitantly [20]. Indeed, it is unclear whether the major driver of immunopathology in BKPyVAN is through viral cytopathic effects, bystander damage mediated by infiltrating BKPyV‐specific lymphocytes, or responses directed against graft alloantigens potentiated by the presence of BKPyV. A greater understanding of what constitutes a protective versus pathogenic immune response may provide key insights toward addressing these challenges and could reveal novel targets for therapeutic intervention.

In this review, we have taken a broad approach to identify and summarise the existing literature pertaining to cellular immune responses generated during BKPyV infection, focussing specifically on KTRs. We consider how the phenotype, functionality, and specificity of BKPyV‐specific T‐cell responses determine the balance between viral control and immunopathology and how these insights may contribute to the development of approaches to predict outcomes and individualize treatment. We also explore the emerging role of natural killer (NK) cells in BKPyV infection, before examining the extent to which peripheral cellular responses correlate with those in the allograft. Lastly, we identify areas where future research may provide key insights to inform the development of novel therapeutic options that would transform how we manage patients after kidney transplantation.

## 2 Method

To identify the relevant literature, we searched Medline and EMBASE using free text terms and Medical Subject Headings which included “BK Virus”, “Polyomavirus infections”, “Organ transplantation” and “Kidney Transplantation”. Searches were limited to English language articles and to human studies. Identified articles were manually de‐duplicated in EndNote and then uploaded to Rayyan (https://rayyan.ai/) to facilitate initial screening of titles and abstracts [21]. Mohammed Al‐Talib and Anna Skaria screened all identified articles independently, with disagreements resolved by discussion. We included original research articles that described any aspect of the immune response against BKPyV in KTRs. Articles referring to other organ transplantation or hematopoietic stem cell transplantation were excluded. Studies focussing on immune responses to other polyomaviruses, including JC polyomavirus (JCPyV) were excluded unless BKPyV was additionally examined. This search strategy identified 207 studies, which were supplemented by relevant studies identified through backward citation searching.

## 3 Protective Role of Virus‐Specific T Cells in BKPyV Infection

The earliest evidence for the role of BKPyV‐specific T cells in the control of BKPyV replication was provided by Comoli et al who described the capacity of autologous dendritic cells pulsed with inactivated BKPyV to stimulate proliferation and interferon−γ (IFN‐γ) production by peripheral blood mononuclear cells (PBMCs) ex vivo from both healthy subjects and pediatric KTRs with active BKPyV infection [22]. More compelling evidence from this group came the following year, with BKPyV‐specific cellular immunity, as measured by IFN‐γ enzyme‐linked immunospot (ELISPOT) assays, examined retrospectively in a cohort of 18 KTRs [23]. In this study, the most striking finding was the absence of a detectable peripheral BKPyV‐specific IFN‐γ^+^ T cell response among five patients at the time of BKPyVAN diagnosis, and the later emergence of this response following immunosuppression reduction in two of these patients coinciding with viral clearance and stabilization of graft function. Subsequent studies employing specific viral epitopes [24, 25, 26] or overlapping peptide pools encompassing some [27, 28, 29], or all [16, 30–35], immunogenic BKPyV antigens (small T [ST], large T [LT], viral capsid proteins 1–3 (VP1, VP2, and VP3)) have further characterized these peripheral T cell responses through either ELISPOT assays or intracellular cytokine staining and flow cytometry. Together these studies indicate that active BKPyV DNAemia and/or BKPyVAN are associated with abrogated BKPyV‐specific T cell responses and that viral control correlates with the development of detectable IFN‐γ secreting BKPyV‐specific T cells. Similar techniques have been used to explore the relationship between pre‐existing BKPyV‐specific T cells and the risk of BKPyV DNAemia after transplantation, with mixed findings with regard to their ability to mediate protection from viral replication (discussed further below) [36, 37, 38].

Furthermore, peripheral blood dendritic cell deficiency both pre‐ [39] and post‐transplantation [40] has been associated with a risk of BKPyV reactivation and BKPyVAN, presumably at least in part due to impaired priming of cellular immune responses.

### 3.1 Phenotype and Functionality of Protective BKPyV‐Specific T Cells in KTRs

Unlike IFN‐γ ELISPOT assays, studies employing intracellular cytokine staining and flow cytometry allow for the distinction between CD4^+^ and CD8^+^ T cells, as well as deeper phenotyping and study of cytokine co‐expression to determine which specific T cell subsets may be key in determining the course of BKPyV infection.

Polyfunctional CD4^+^ T cells co‐producing IL‐2, IFN‐γ, and tumor necrosis factor‐alpha (TNF‐α) have been implicated in viral control. A retrospective study of 48 KTRs, of whom 19 had a history of BKPyVAN and the remainder had either transient or no BKPyV DNAemia, identified a greater incidence of patients with IL‐2/IFN‐γ/TNF‐α triple‐producing BKPyV‐specific CD4^+^ T cells among those without a history of BKPyVAN [31]. This was despite patients with a history of BKPyVAN having generally higher magnitude T‐cell responses. Building on this, a study including 27 KTRs with a history of BKPyV DNAemia, of whom 16 had rapid recovery (resolution within 3 months), demonstrated that KTRs with a history of rapid viral clearance had higher frequencies of these triple‐cytokine producing CD4^+^ T cells than those with prolonged recovery, with detectable increases at timepoints of viral load decline and viral clearance, further implicating these cells in viral control [32]. Similarly, an increase in triple‐cytokine producing CD4^+^ T cells associated with viral clearance was observed in a study of 37 KTRs, of whom 27 had a history of BKPyV DNAemia, whereby PBMCs were stimulated with 15mer overlapping peptide pools and then examined by multiparameter flow cytometry. These authors used co‐expression of activation markers such as CD137, CD154, and Granzyme‐B to detect BKPyV‐specific T cells, rather than IFN‐γ production, as these markers may be less influenced by immunosuppressive drugs than cytokine expression [34]. Of note, an increase in cytolytic CD4^+^ T cells accompanied viral clearance in this study, suggesting a direct effector function for CD4^+^ T cells in BKPyV control.

The role of polyfunctional CD8^+^ T cells is less clear, with some studies described above observing no association with viral control and/or clearance [32, 34]. While this may reflect these responses being less clinically relevant, it is important to note many studies examining BKPyV‐specific T‐cell responses have utilized 15mer peptide pools to stimulate PBMCs prior to analysis. While expected to capture both CD4^+^ and CD8^+^ T cell responses, 15mers are larger than peptides typically bound by major histocompatibility complex (MHC) Class I in vivo, and as such these studies may have underestimated the contribution of CD8^+^ T cell responses in these cohorts. This has been demonstrated in studies of HIV vaccine efficacy [41]. Indeed, studies using 9mer peptide pools have identified robust relationships between the magnitude of CD8^+^ T cell responses and viral clearance among a cohort of 28 KTRs with a history of DNAemia [35] and, separately, modest proportions of triple‐cytokine‐producing CD8^+^ T cells among 11 KTRs with low‐level viral replication (less than 10^4^ copies/mL) [26]. Adding further complexity, Schaenman et al reported an 11‐fold increase of double (two of IL‐2, IFN‐γ and/or TNF‐α) and triple‐cytokine producing CD8^+^ T cells among 10 KTRs who controlled DNAemia within 3 months versus eight non‐controllers after stimulating PBMCs with 15mer peptide pools [33]. Interestingly, no differences were observed in multiple‐cytokine‐producing CD4^+^ T cell frequencies between groups in this study, which could suggest that these responses are more important in maintaining viral control, while CD8^+^ T cells may be more important in mediating viral clearance.

BKPyV infection outcome may also be explained by clonotype diversity and the degree of T cell functionality. Stervbo et al examined factors that may explain the time taken to achieve viral clearance, which ranged from weeks to years in their cohort of seven KTRs with sustained BKPyVAN DNAemia, of whom four had developed this within 5 months of transplantation [42]. Their approach combined multiparameter flow cytometry and next‐generation sequencing‐based T‐cell receptor (TCR) clonotype profiling to track the functional activity of specific peripheral T‐cell clones over the course of BKPyV resolution. Low clonotype diversity and expression of exhaustion markers PD‐1 and TIM3 at the time of initial viral load decline were significantly correlated with prolonged clearance time. Interestingly, the magnitude or phenotypic characteristics of CD4^+^ and CD8^+^ T cell responses—such as multiple‐cytokine‐producing ability—at the peak of viral load were not correlated with viral clearance. This last finding appears to conflict with previous studies associating polyfunctional T‐cell responses with viral control [32, 33] but may support the theory that different cellular immune mechanisms are involved in establishing initial viral control versus mediating viral clearance. This study also highlighted one patient who was able to rapidly control viral replication despite low TCR diversity. This appeared to be overcome by BKPyV‐specific T cells with high functional fitness and circulating at high magnitude. Given this study was relatively small and included patients with large differences in time taken to achieve viral clearance, further studies of larger cohorts and considering TCR repertoire avidity may shed further light on the importance of these factors.

### 3.2 Antigen Specificity of the Developing T‐Cell Response May Reflect Course of BKPyV Infection

**FIGURE 2:**
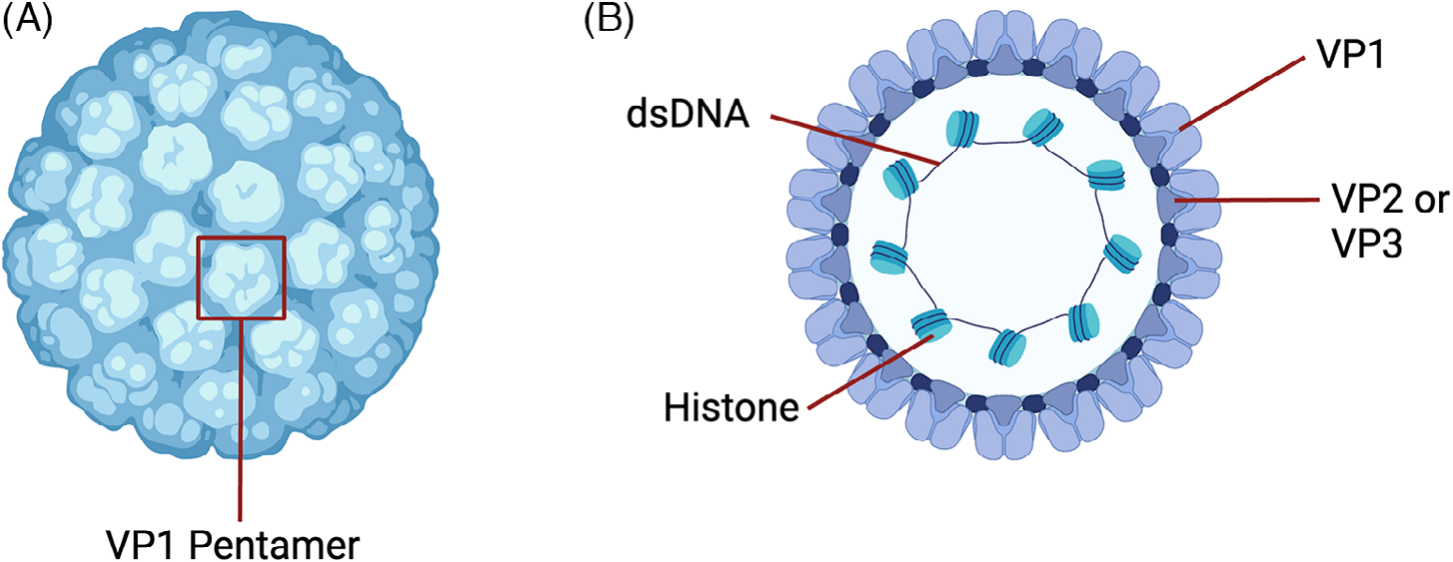
Schematic representation of the BK polyomavirus virion. (A) The BK polyomavirus (BKPyV) capsid consists of 360 VP1 monomers arranged as 72 pentamers in a *T = 7d* icosahedral structure 40–45 nm in diameter. (B) Each VP1 pentamer is associated with a single copy of either minor capsid protein VP2 or VP3 at the internal surface.

The considerable inter‐ and intra‐patient variability in cellular immune responses to different BKPyV antigens over the time course of infection may point to differing modes of action of T cells that target distinct viral antigens (Figure 2). Among 42 KTRs with decreasing or past BKPyV DNAemia, IFN‐γ responses against VP1 were greater than those against LT and were predominantly CD4^+^ T cells, while CD8^+^ T cells were more frequently directed against LT [27]. However, few studies have examined differential CD4^+^ and CD8^+^ T cell responses against all viral antigens separately. One retrospective study that did examine this included nine KTRs with a history of BKPyVAN and found the capsid protein VP3 elicited the highest frequencies of BKPyV‐specific T cells, although antigens from all five immunogenic BKPyV proteins could elicit detectable CD4^+^ and CD8^+^ T cell responses [31]. CD4^+^ T cell responses were, however, dominant, with 74% of patients having simultaneously detectable responses to all five proteins in this study. In contrast, no patient in this study had CD8^+^ T cells targeting all five proteins simultaneously. Another study examined responses against single and mixed BKPyV protein peptide pools in a cohort of 39 KTRs stratified into three groups: history of BKPyV DNAemia lasting greater than 3 months, history of DNAemia lasting less than three months, and no history of DNAemia [32]. Higher frequencies of IL‐2, IFN‐γ, and TNF‐α producing CD4^+^ T cells targeting individual BKPyV structural proteins (VP1–VP3) compared to regulatory proteins (ST and LT) were detected in patients with a history of prolonged DNAemia. However, CD4^+^ T cells targeting ST and LT were detected at comparable frequencies to those against VP1‐VP3 among KTRs in the other two groups. CD8^+^ T cell responses were again lower in magnitude generally, with VP2 eliciting the greatest response in all groups. As discussed previously, these differences may in part be explained by the 15mer peptide pools preferentially stimulating CD4^+^ T cells ex vivo. Indeed, the requirement for ex vivo stimulation to detect BKPyV‐specific T cells makes it difficult to infer whether CD4^+^ or CD8^+^ T cells targeting specific viral antigens influence infection outcomes in vivo. However, these findings offer some support to the hypothesis that T cells targeting regulatory protein antigens may be important in achieving viral clearance. This was suggested in another study that aimed to unpick viral load dynamics and the development of peripheral CD4^+^ and CD8^+^ T cell responses, measured by IFN‐γ ELISPOT, against 15mer peptide pools in six KTRs sampled longitudinally from diagnosis of BKPyVAN to subsequent viral clearance [43]. Despite the wide range of times needed to achieve viral clearance (117 to 1744 days), commonly observed patterns after immunosuppression reduction included earlier development of anti‐VP responses, and the emergence of anti‐ST/LT responses, associated with faster viral load decrease, echoing previous studies [16, 27]. Integrating these data into mathematical models, these authors found most evidence supporting the hypothesis that anti‐VP responses predominantly reduce virion production, whereas anti‐ST/LT responses accelerate the killing of infected cells. In this model, anti‐ST/LT responses were key to achieving rapid and sustained viral clearance. Further experimental evidence is required to corroborate these predictions, and this model was not supported by all patients studied, reflecting the complexity of the BKPyV‐specific cellular immune response. Further characterization of the antigen specificity of T cell response against BKPyV, and how the evolution of these responses correlates with viral control, may support the selection of key immunodominant epitopes—of which nearly 100 from BKPyV regulatory proteins have already been identified [25, 35]. Excitingly, immunodominant 9mer‐stimulated CD8^+^ T cell responses at 6 and 12 months post‐transplantation from 28 KTRs correlated with clearance of BKPyV DNAemia [25]. These T cells were expandable in vitro, offering the potential to be harnessed in the development of autologous adoptive T cell therapies, in addition to informing the design of vaccines. It is worth noting however that, despite being highly conserved, amino acid‐exchanges have been identified within immunodominant 9mer LT epitopes that are predicted to alter human leukocyte antigen (HLA)‐A/HLA‐B presentation [44], and one such variant elicited lower magnitude CD8^+^ T cell IFN‐γ responses in two healthy individuals after ex vivo stimulation, suggesting a possible mechanism for immune escape in KTRs that warrants further investigation [44].

### 3.3 The Role of Pre‐Transplantation BKPyV‐Specific T Cells in Determining BKPyV Outcome is Not Established

While there is considerable literature pertaining to pre‐transplantation serostatus and BKPyV risk [45, 46, 47, 48, 49], the study of the contribution of pre‐existing cellular immunity has been relatively limited, and the results conflicting. BKPyV‐specific T cell responses were measured by IFN‐γ ELISPOT from PBMCs in 108 KTRs sampled before, and 1, 2, and 3 months after transplantation, following ex vivo stimulation using separate overlapping peptide pools spanning VP1 and LT [36]. The development of early‐onset (<30 days after transplantation) BKPyV DNAemia was associated with a significant decrease in LT‐specific T cells from pre‐transplantation to one month post. Interestingly, 11/16 KTRs with early‐onset DNAemia had measurable LT‐specific T cells prior to transplantation. In contrast, only 7/92 KTRs without BKPyV replication had similar detectable responses, and these patients did not experience a dramatic decline in LT‐specific T cell numbers at one month. These findings may indicate that KTRs with increased susceptibility to immunosuppression may be predisposed to a loss of BKPyV‐specific immunity that results in viral replication. However, in a separate study of 31 consecutively enrolled KTRs (seven of whom developed BKPyV DNAemia), no relationship was observed between BKPyV‐specific CD4^+^ T cells pre‐transplantation and subsequent DNAemia [37]. Additionally, nearly all patients had detectable BKPyV‐specific T cells pre‐transplantation as measured by IFN‐γ ELISPOT. Likewise, a small study of 21 KTRs reported VP1‐specific CD4^+^ and CD8^+^ T cells were detectable pre‐transplantation in 95% and 76% of participants respectively [38]. In this study, regression modeling was used to suggest both BKPyV‐specific CD4^+^ and CD8^+^ T cell responses increased monthly among patients with DNAemia, although given only three patients in the study developed DNAemia, these findings must be interpreted with considerable caution. Why these studies present such contradictory findings is unclear, although the profound differences in proportions of patients with detectable BKPyV‐specific T cells pre‐transplantation by IFN‐γ ELISPOT suggests systematic differences in the stimulation protocols used, positive and negative control cut‐offs, and/or the stability of the assayed PBMCs. Indeed, a systematic review and meta‐analysis of studies employing IFN‐γ ELISPOT assays to measure BKPyV immune responses highlighted the variation in thresholds across studies [50]. Larger, standardized prospective studies measuring T‐cell responses and BKPyV replication both prior to, and at regular timepoints after, transplantation may shed greater light on the relationship between pre‐transplant cellular immunity and subsequent BKPyV risk.

## 4 Clinical Applications of BKPyV‐Specific T‐Cell Response Measurement

With numerous studies tying clearance of BKPyV DNAemia with an increase in BKPyV‐specific T cells, the question of whether these observations can be adopted into tools to prognosticate BKPyV outcome remains. This may be of real importance: overzealous immunosuppression reduction may precipitate rejection, whereas persistent high‐level DNAemia may result in BKPyVAN and irreversible graft damage. Data from a retrospective analysis of T cell function in a patient who ultimately lost their graft following episodes of BKPyVAN and TCMR supported the use of functional immune monitoring as a potential tool to guide management [51]. More definitively, Ahlensteil‐Grunow et al prospectively measured BKPyV‐specific T cell responses in 32 pediatric KTRs after diagnosis of BKPyV DNAemia [18]. While the initial viral load had no impact on the duration of DNAemia, they found a negative correlation between the frequency of IFN‐γ^+^ BKPyV‐specific CD4^+^ and CD8^+^ T cells at, or soon after, diagnosis and the subsequent duration of DNAemia. Thresholds of ≥0.5 cells/µL for BKPyV‐specific CD4^+^ T cells and/or ≥0.1 cells/µL for BKPyV‐specific CD8^+^ T cells predicted transient, self‐limited DNAemia not requiring immunosuppression reduction (positive predictive value 1; negative predictive value 0.86). Whereas CD8^+^ T cell responses were transient, CD4^+^ T cell responses persisted even after clearance of DNAemia, suggesting these could also be used as a follow‐up parameter to guide ongoing management. Larger studies including adult KTRs and across centers with varying induction and/or maintenance immunosuppression regimens, are required to validate these cut‐offs. Ultimately, randomized controlled trials will be needed to determine whether BKPyV management based on T cell monitoring results in better graft outcomes versus standard of care.

## 5 NK Cells: An Unknown Entity in BKPyVAN

**FIGURE 3:**
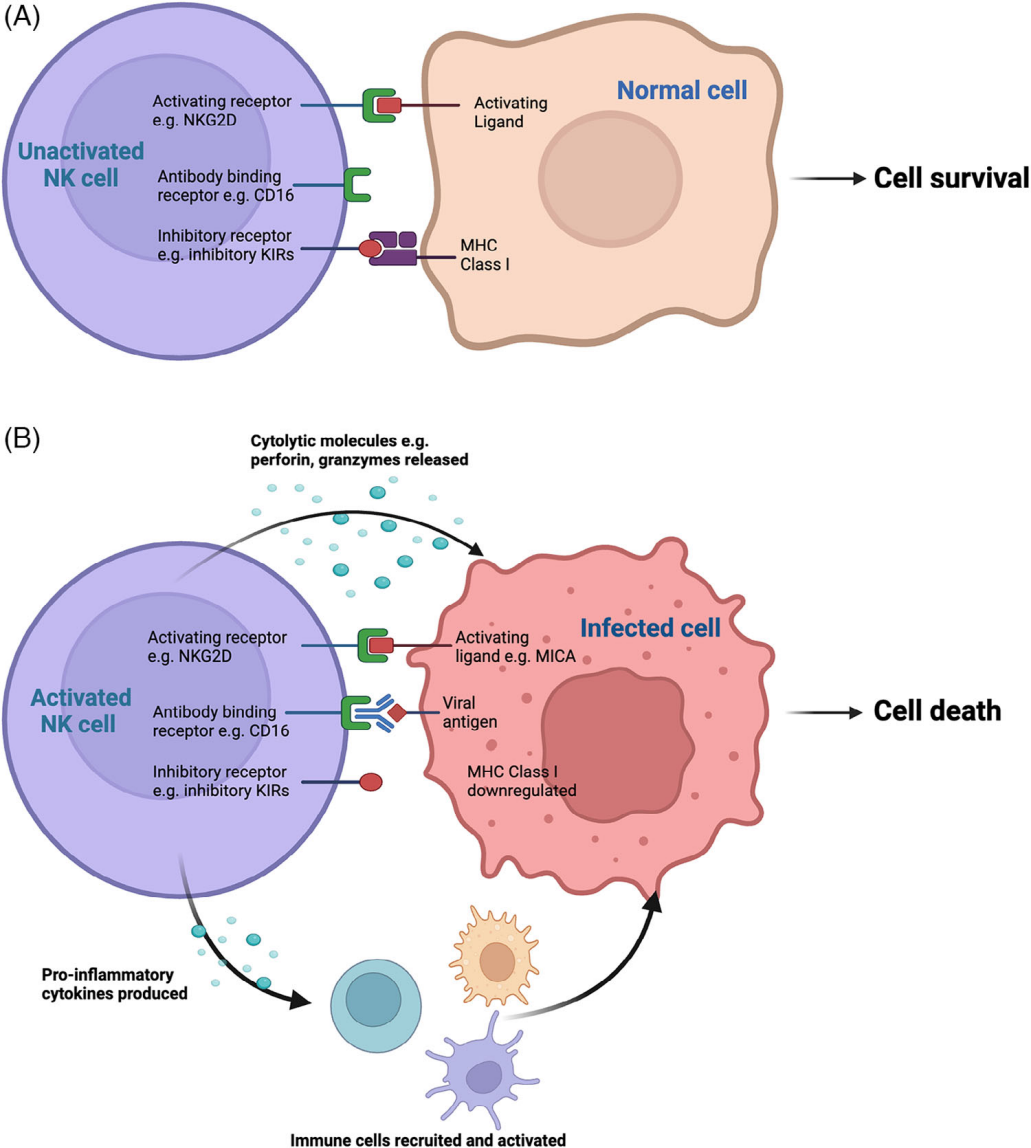
Overview of natural killer (NK) cell recognition of healthy and virus‐infected cells. (A) Balance of inhibitory and constituent activating signals from health cells prevents NK cell activation. (B) NK cell targeting of an infected cell through multiple mechanisms including upregulation of activating ligands on the infected cell; CD16‐mediated antibody‐dependent cellular cytotoxicity and loss of inhibitory killer‐cell immunoglobulin‐like receptor (KIR) signaling due to down‐regulation of major histocompatibility complex (MHC) class I expression on the virus‐infected cell (so‐called “missing‐self”).

NK cells are a heterogenous population of CD3^−^ CD56^+^ lymphocytes that target infected, transformed, and stressed cells and are key contributors to antiviral immunity [52]. They respond to virally infected cells through a complex array of activating and inhibitory receptors, the aggregated signaling through which determines whether the target cell is killed (Figure 3). Mechanisms of NK cell activation include antibody‐dependent activation via the Fc‐receptor CD16, activation following detection of cell‐stress molecules triggered by viral infection (e.g., ligands for the Natural Killer Group 2D [NKG2D] C‐type lectin‐like receptors), and, importantly, control through the highly polymorphic killer‐cell immunoglobulin‐like receptors (KIRs) [53], which detect alterations in MHC class I expression induced by some viruses. Key work implicating NK cells in BKPyV control came from Trydzenskaya et al, who genotyped 158 KTRs (48 of whom had biopsy‐proven BKPyVAN) to identify KIR haplotypes and their association with BKPyV outcome [54]. KTRs carrying lower numbers of activating KIRs, particularly those lacking KIR3DS1, had a greater likelihood of developing BKPyVAN (odds ratio 0.4; 95% confidence interval 0.1–0.9; *p* = 0.02). This was independent of immunosuppressive burden. Further studies have reported greater BKPyV DNAemia/BKPyVAN risk among KTRs with lower numbers of activating KIRs [55] and among KTRs carrying the inhibitory KIR2DL3 [56]. Although associations with specific KIRs have not been validated, these findings indicate that some KTRs may have a genetic predisposition to BKPyV infection and BKPyVAN mediated through NK cell functionality.

Building upon this, the observed association between some HLA alleles (reviewed elsewhere [57]) and risk and/or protection from BKPyV may be partly explained by these HLAs serving as ligands for KIRs. There is the greatest evidence for the role of HLA‐Cw7, with multiple studies suggesting its expression in donors and/or the recipient is associated with a lower risk of BKPyV DNAemia and BKPyVAN [55, 58, 59]. The combination of donor HLA‐Cw7 negativity and recipient lack of activating KIRs was also found to be more common among 23 KTRs with BKPyVAN [55]. It is worth noting, however, that while these associations may be mediated through NK cells, the presentation of specific viral epitopes by these HLAs to CD8^+^ T cells must be taken into account, particularly as BKPyV may escape immune recognition by acquiring mutations within predicted cognate HLA‐C‐bound viral peptides [60].

Beyond HLA and KIRs, the role of NK cells in BKPyV immunity has been inferred from studies of other NK cell ligands. Tonnerre and colleagues investigated the role of MHC class I–related chain A (MICA)—a ligand for NKG2D expressed in tubular epithelial cells—upon BKPyV infection among 144 transplant donor/recipient pairs [61]. They observed a lower incidence of BKPyV reactivation in recipients of grafts from donors expressing the variant MICA A5.1. These authors concluded that this variant is protective against BKPyV, perhaps due to its interaction with NKG2D. Similar associations with BKPyV infection outcomes have been identified with polymorphisms in HLA‐E, a non‐classical MHC molecule that can inhibit and/or activate NK cells through NKG2A/C receptors [62]. Furthermore, laboratory studies indicate that BKPyV may evade NK cell recognition through the BKPyV‐derived microRNA bkv‐miR‐B1‐3p, which downregulates UL16‐binding protein 3 (ULBP3) expression, another ligand for NKG2D [63].

Despite this, our understanding of the role of NK cells in controlling BKPyV and/or mediating tissue damage in BKPyVAN remains limited. Studies exploring the relationship between HLA/KIRs and BKPyV are relatively small, and positive associations reported could reflect type 1 error because of multiple tests. Clinically, the phenotype and functionality of peripheral NK cells and how these relate to BKPyV infection time course after kidney transplantation have not been thoroughly explored. A single‐center study recently reported a higher proportion of IFN‐γ^+^ VP1‐responsive NK cells at 1 month after transplantation in 18 KTRs who developed either BKPyV viruria or DNAemia [64], however, the findings may have been skewed by a single patient in this group with very high NK cell levels. In addition, mechanistic evidence of NK cells mediating protection against BKPyV is lacking, and indeed NK cells may contribute to immunopathology in BKPyVAN: NK cell‐associated transcripts are up‐regulated in BKPyVAN biopsies [65], and one clinical study including 43 KTRs with BKPyV viruria reported that BKPyV replication was more frequent in KTRs with more responsive NK cells [66]. Furthermore, the role of adaptive NK cell subsets (expressing markers such as Immunoglobulin‐like transcript 2 [LIR1 or LILRB1], NKG2C, and CD57) which are associated with control of cytomegalovirus (CMV) in KTRs [67, 68, 69], has not been examined in BKPyV. Further prospective studies involving large cohorts of KTRs, supported by basic sciences approaches, are needed to elucidate the roles of NK cells in BKPyV infection.

## 6 A Double‐Edged Sword? BKPyV‐Specific T Cells Within the Allograft

While the study of BKPyV‐specific cellular immune responses in peripheral blood has provided key insights, the immunopathology of BKPyVAN occurs within the allograft. Here, there is an apparent contradiction between the fact that the development of detectable BKPyV‐specific cellular immunity in the periphery correlates with viral control and is a precursor to the resolution of BKPyVAN, whereas BKPyVAN is largely indistinguishable from TCMR histologically. This raises the question of whether BKPV‐specific cellular responses in the allograft contribute, directly or indirectly, to allograft damage. Clearly, a greater understanding of the nature and specificity of intragraft responses in BKPyVAN is needed.

One approach is immunohistochemistry, which has been used to identify FoxP3 positivity in allografts that increased with BKPyVAN severity [70]. The clinical significance of these presumably regulatory T cells is unknown but may represent a futile response to limit immunopathology or, conversely, may be mediating aberrant suppression of antiviral effector responses that would otherwise promote viral clearance.

Intragraft responses have also been examined using tetramer staining of CD8^+^ T cells eluted from allografts, which in a study of two patients with BKPyVAN revealed VP1‐specific cells that were enriched 10^5^ times compared to peripheral blood but shared a similar CD45RA^−^CD27^+/−^ T_EM_ phenotype [26]. These cells also had high expression of CD69 and CD103, designating them as tissue‐resident memory T cells (T_RM_), and showed little Granzyme‐B positivity. A recent analysis of tissue‐resident lymphocytes in explanted kidney allografts also identified BKPyV‐specific T_RM_ of both donor and recipient origin [71]. Whether donor and recipient‐derived T_RM_ have distinct functionality, and why these high‐frequency virus‐specific T_RM_ cells seem unable to exert viral control, requires further study.

Another approach has been to use next‐generation sequencing of genomic DNA to define the TCR repertoire of T cell infiltrates in renal allografts with BKPyVAN and TCMR [72]. Using this method, the authors could then define T‐cell clones that expanded when PBMCs were stimulated by viral or mismatched HLA peptides, and subsequently track these clones in biopsy specimens. Although the analysis was restricted to responses to HLA antigens or BKPyV epitopes (LT only), and direct antigen presentation during the ex vivo stimulations was not evaluated, a striking finding was that alloreactive T cell clones were around 10 times more abundant than BKPyV‐reactive clones in BKPyVAN biopsy specimens. The phenotype of these clones was not evaluated, but these findings suggest the immunopathology of BKPyVAN could be driven more by recruitment of alloreactive or bystander T cells, rather than BKPyV‐specific T cells.

Analyses of intragraft gene expression have also demonstrated differences in immune cell activity in BKPyVAN. For example, Lubetzky et al reported upregulation of several pathogenesis‐based transcript sets including cytotoxic T cell and NK cell activity [65]. However, many of these transcript‐based studies have demonstrated striking overlap with TCMR [65, 73, 74]. Adam et al addressed this issue elegantly by comparing gene expression in a discovery cohort of native kidney BKPyVAN versus pure TCMR using the NanoString platform, however, index biopsy gene expression was not predictive of the outcome at 6 months, and the BKPyVAN immune gene set identified failed to adequately discriminate allograft BKPyVAN and TCMR [75]. Although perhaps unsurprising given their similar histological appearances, this points to the current limitations of these techniques in fully discriminating the BKPyV‐specific T cell response from cognate T cell‐mediated inflammation directed against graft alloantigens. However, as molecular approaches continue to progress, already we are gaining insight into how they may be used in the future to support clinical decision‐making. For example, the genome‐wide microarray platform utilized in the Molecular Microscope Diagnostic System (MMDx) identified diffuse tubulitis and a positive TCMR classifier in BKPyVAN as suggestive of super‐imposed alloimmune TCMR that may otherwise have been missed histologically [19].

## 7 Future Directions and Challenges

A major limitation of much of the research to date is that most studies have been relatively small and conducted in single centers, often comparing patient samples at variable timepoints before, during, and after BKPyV DNAemia and/or BKPyVAN. This may in part explain the lack of consistent results reported in studies of BKPyV cellular immunity. Larger, ideally multi‐center, studies using standardized blood collection regimens beginning prospectively prior to transplantation are needed to fully evaluate the peripheral anti‐BKPyV cellular immune response and its temporal relationship with infection. More sensitive techniques are required to identify and characterize BKPyV‐specific T cells without the need for ex vivo stimulation and expansion which, although necessary due to the low frequency at which these cells normally circulate, may be expected to alter the phenotypic characteristics of these cells. Beyond this, there are a number of technical challenges in measuring BKPyV‐specific cellular immunity, including the integrity of cryopreserved PBMCs, choice of peptides for ex vivo stimulation, and epitope mutational escape, that have been thoroughly reviewed recently [76]. Epitope specificity is also a consideration, given that JCPyV shares approximately 70% sequence homology with BKPyV and can rarely produce a clinical picture similar to BKPyVAN in KTRs [77]. In non‐immunocompromised individuals, CD8^+^ T cells that recognize HLA‐A2‐restricted epitopes shared by BKPyV and JCPyV have been identified in peripheral blood, raising the possibility that infection with one of these viruses may confer cross‐protective immunity against the other [78]. Supporting this, some immunodominant BKPyV 9mer epitopes share common sequences with JCPyV [25]. Whether this cross‐reactivity could affect assays of BKPyV cellular immunity by producing non‐specific responses after ex vivo stimulation is unclear, and it is not known whether pre‐existing cellular immunity against JCPyV may confer protection against BKPyV, or vice versa.

In addition, the extent to which BKPyV‐specific cellular immunity measured from peripheral blood actually correlates with the immunopathology of BKPyVAN is unknown. Furthermore, is the absence of detectable peripheral BKPyV‐specific T cells during BKPyVAN a reflection of the genuine lack of this response in the immunosuppressive context, or due to sequestration of these T cells in the renal tissue? If it is the latter, to what extent are these T cells contributing to tissue damage versus resolution of infection? Adding a further layer of complexity, the interplay between T cells and other components of the innate cellular and humoral immune response is undefined. Although beyond the scope of this review, humoral immunity appears to play some role in determining the risk of development of BKPyV DNAemia, with seronegative recipients of allografts from seropositive donors at higher risk [79]. Furthermore, the use of intravenous immunoglobulin in the treatment of BKPyVAN may suggest a role for humoral immunity in mediating viral clearance [80], however robust efficacy data from clinical trials is lacking and the development of BKPyV‐specific IgG, unlike T cells, in patients with BKPyV DNAemia does not appear to correlate with viral clearance [16].

One key area that warrants investigation is the utility of monitoring BKPyV‐specific cellular immunity to prognosticate and, more importantly, guide immunosuppression management in KTRs with BKPyVAN. Validating cut‐offs established in previous studies is an important first step, as well as exploring whether integration with other markers of innate and/or humoral immunity shows greater sensitivity and specificity. Ultimately, any assays of T cell function would need to be standardized, easy‐to‐use and readily available and whether commercial immune function assays specific for BKPyV, such as those for CMV [81] or cellular immune function generally [14], are on the horizon remains to be seen.

The observations that peripheral BKPyV‐specific T cell responses develop in association with BKPyV control have also fed interest in using either donor‐derived or third‐party virus‐specific T cells (VSTs) as adoptive immunotherapy [82, 83, 84, 85, 86]. Perhaps the most promising platform was Posoleucel (ALVR105; AlloVir), an off‐the‐shelf multi‐virus specific therapy with favorable safety and efficacy data from phase 2 trials in both allogeneic hematopoietic stem cell [87] and renal transplantation [88]. However, three phase 3 studies of Posoleucel outside of renal transplantation were recently abandoned at interim analyses for futility, highlighting that a greater understanding of what constitutes a protective T‐cell response, particularly within infected tissues, is required. Further characterization of broadly reactive and highly immunogenic TCR epitopes will facilitate the design of more effective VSTs, in addition to supporting the development of vaccines. Immunotherapies harnessing NK cells, as have been demonstrating promising results in cancer [89], may also be explored if a protective role for NK cells can be defined.

Advances in molecular approaches, for example, single‐cell RNA sequencing of allograft biopsies taken at different stages of BKPyV infection, may provide detailed insights into intragraft gene expression and the array of T‐cell phenotypes and networks that mediate viral control and immunopathology. These techniques may also reveal fundamental differences between BKPyVAN and TCMR that could help guide decision‐making in patients with equivocal histological findings. However, the study of intragraft pathology over the time course of BKPyVAN has a number of inherent challenges. A kidney biopsy is not without risk, and the patchy nature of the disease necessitates sampling of multiple cores to establish a diagnosis, with a resultant increased risk of complications. Discordant findings between two cores taken from the same allograft have been reported in 36.5% of cases [90], and it is therefore possible that a core taken specifically for research purposes may miss the infected foci. Development of safer tissue‐sampling techniques such as fine needle aspiration, and better correlation of findings from allograft biopsies with time‐matched blood and, potentially, urine samples may also provide a route towards minimally invasive diagnosis and monitoring or BKPyVAN. Furthermore, BKPyVAN may be ideally suited to spatial transcriptomics analyses to examine gene expression at specific sites of immunopathology from archival tissue samples. Already, these technologies are being applied to reveal the complexities of rejection in unprecedented detail [91, 92]. The establishment of biobank studies recruiting a diverse array of patients from pre‐transplantation and obtaining and storing relevant samples (such as whole blood, PBMCs, plasma, urine, and tissue) for analysis at pre‐defined timepoints over the course of transplantation will be an invaluable resource for researchers.

## 8 Conclusions

BKPyV‐specific T cells appear to play a key role in clearing BKPyV in KTRs, with the emergence of detectable peripheral CD4^+^ and CD8^+^ T cell responses temporally correlating with viral control, and greater magnitude responses generally associated with rapid viral clearance. Tracking these responses over the time course of BKPyV infection may facilitate better prognostication and guide individualized immunosuppression reduction strategies. The role of other components of cellular immunity, in particular NK cells, requires further investigation. Greater characterization of intragraft cellular responses, perhaps using novel bioinformatics approaches, may reveal the balance between viral control versus immunopathology, and identify markers to distinguish BKPyVAN from TCMR. Although adoptive cellular immunotherapies may be some way from clinical use, building our knowledge of the cellular immune response against BKPyV may drive the development of much‐needed therapeutic interventions.

## Conflicts of Interest

The authors declare no conflicts of interest.

## Data Availability

Data sharing is not applicable to this article as no datasets were generated or analyzed during the current study.
